# Correlates of preferences for home or hospital confinement in Pakistan: evidence from a national survey

**DOI:** 10.1186/1471-2393-13-137

**Published:** 2013-06-24

**Authors:** Sajid Amin Javed, Muhammad Danish Anjum, Waqas Imran, Azad Haider, Ayesha Shiraz, Farzana Shaheen, Muhammad Iftikhar ul Husnain

**Affiliations:** 1 Department of Economics, Pakistan Institute of Development Economics, Quaid-e-Azam University Campus, Islamabad 44000, Pakistan; 2 IQRA University, Islamabad, House # O/1026-B, Mohallah Huri Pura Ghazi Roadi, Rawalpind, Pakistan; 3 The Islamia University of Bahawalpur, H-268, Gali-34, G-10/1, Islamabad 44000, Pakistan; 4 COMSATS Institute of Information Technology, Islamabad 44000, Pakistan; 5 National Institute of Population Studies, 12-A Capital Inn Building, G-8 Markaz, Islamabad 44000, Pakistan; 6 Federal Urdu University of Arts, Science and Technology, Islamabad 44000, Pakistan

**Keywords:** Reproductive health, Place of delivery, Pakistan, Logistic regression

## Abstract

**Background:**

Despite the pregnancy complications related to home births, homes remain yet major place of delivery in Pakistan and 65 percent of totals births take place at home. This work analyses the determinants of place of delivery in Pakistan.

**Methods:**

Multivariate Logistic regression is used for analysis. Data are extracted from Pakistan Demographic and Health Survey (2006–07). Based on information on last birth preceding 5 years of survey, we construct dichotomous dependent variable i.e. whether women deliver at home (Coded=1) or at health facility (coded=2).

**Results:**

Bivariate analysis shows that 72% (p_≤_0.000) women from rural area and 81% women residing in Baluchistan delivered babies at home. Furthermore 75% women with no formal education, 81% (p_≤_0.000) women working in agricultural sector, 75% (p_≤_0.000) of Women who have 5 and more children and almost 77% (p_≤_0.000) who do not discussed pregnancy related issues with their husbands are found delivering babies at home. Multivariate analysis documents that mothers having lower levels of education, economic status and empowerment, belonging to rural area, residing in provinces other than Punjab, working in agriculture sector and mothers who are young are more likely to give births at home.

**Conclusion:**

A trend for home births, among Pakistani women, can be traced in lower levels of education, lower autonomy, poverty driven working in agriculture sector, higher costs of using health facilities and regional backwardness.

## Background

Reproductive health problems remain leading cause of death of women of childbearing age worldwide (PDHS, 2006–07). It is alarming that after every 20 minutes a mother dies in Pakistan due to pregnancy related complications (Pakistan Bureau of Statistics). Maternal Mortality Rate in Pakistan is as high as 276 deaths per 100,000 live births (PDHS, 2006–07). Recently, improved MCH services have been introduced in Pakistan by inducting Lady Health Workers to provide health education and raise awareness in the country (National Institute of Population Studies [1]). However, the slow progress at this front can be, most probably, traced in low emphasis on community participation and inadequate human resource development. Home births remain one of the major causes of maternal deaths (Bhave, [2]) and, according to United Nation Fund for Poverty Alleviation [3], 536,000 women die every year due to pregnancy complications.

Voluminous work, across the globe, has been undertaken to understand the correlates of choice of place of delivery but the issue remains unexplored in Pakistan. To the best of our knowledge, it is first study in this domain and aims at exploring the factors underlying mothers ‘attitude towards making a choice of place for delivering babies.

## Methods

Data are extracted from Pakistan Demographic and Health Survey (PDHS) 2006–07; the second round of worldwide DHS project carried out in Pakistan by National Institute of Population Studies (NIPS). The survey covers a sample of 10,023 ever-married women of reproductive age (15–49 years) selected through multistage clustering sampling from all over the Pakistan using a sampling framework provided by PBS. For the analysis at hand, information from pregnancy, labor and postnatal section of the women questionnaire has been drawn to constitute the target cases (weighted) of last child born in five years preceding the survey (N=5677). Analysis is undertaken by applying bivariate and multivariate logistic regression. There was no need for ethical clearance since the work used secondary analysis of publically available data.

### Variable description

Dependent variable is defined whether the birth took place at home (code=1) or health facility (code=0); health facility includes government health center/mother and child health, other public and private hospital/clinic. Independent variables include socio- economic and demographic characteristics of mothers. Table 1 provides information on construction of variables.

**Table 1:** Variable description

**S.no.**	**Variable**	**Construction**
1	Education of Woman	No Education®, Primary to Middle, Above Middle
2	Education of Husband	No Education®, Primary to Middle, Above Middle
3	Occupation of Mother	Not working®, Agriculture, Sales, and Others
4	Mother Ever Discussed with her husband about where to deliver	Discussed , Not Discussed®
5	Age of Mother (Years)	15 – 24®, 25 – 34, 35 – 49
6	Birth Order (Years)	Less than 3®, 3 – 4, 5 and Above
7	Regions	Punjab®, Sindh, KP and Baluchistan
8	Residence	Urban®, Rural
9	Economic Status	Rich®, Poor, Middle

® Reference Category.

Economic status of the mother is captured through wealth index (poor, middle & Rich class) wherein poor includes (poorer and poorest) while the richer and richest categories are merged in rich. Worth noting, here, is that PDHS 2006–07 used, in collaboration with Measure DHS, the mother organization for conducting Demographic and Health Surveys across the world, Principal Components Analysis (PCA) to construct economic status variable (see section 2.6 of PDHS and http://www.measuredhs.com/pubs/pdf/CR6/CR6.pdf for details). Educational level of women and her husband is divided into three categories namely no educational, primary to middle and above middle. Current age of mother is categorized in 15–24, 25–34 and 35–49 purposefully exhibiting three different period of reproductive age. Similarly occupation of mother is divided into four major categories i.e. no working, agriculture, sales and others. The agriculture category includes self-employed also while sales includes professional, technical, management, clerical, services and household/domestic jobs. Rests of the professions are included in others. Women empowerment is captured through the indicator whether the women discuss with her husband about place of delivery or not where the farmer indicates empowerment.

## Results

### Reasons for delivering babies at home- a descriptive analysis

It is a revealing fact that most of the maternal deaths can be prevented if adequate and timely emergency obstetric care is provided. The WHO safe motherhood program of 1987, emphasizes the importance of access to EmONC, to manage the common causes of obstetric deaths: hemorrhage, obstructed labor, complications due to unsafe abortion, eclampsia, and infection. Thaddeus and Maine [4] put forwarded that a bulk of maternal deaths is associated with home births and is avoidable by creating awareness.

It is evident from Table 2, reporting the reasons for choice of home deliveries amongst Pakistani women, that higher cost remains most important reason for opting home confinement. 29% and 40% of women from urban and rural domain respectively reported that higher cost of delivering baby at health facility lead to rather stay home and deliver baby. In Sindh and Baluchistan provinces 50% and 40% of the mothers give higher costs of delivering baby as a reason not to avail the facility respectively. Most important, however, is higher rate of women who do not find necessary to deliver at health facility and the proportions reached to 46% and 40% respectively for rural and urban mothers respectively. While in Punjab province 49% mothers find delivering at health facility unnecessary. Interestingly the percentage is higher among women belonging to rich status and 57% of them deliver baby at home by choice and think that delivering at health facility is not necessary.

**Table 2:** Percent distribution of women, five years preceding the survey, having deliveries at home

**Characteristics**	**Reasons for delivering at home**					**N**
	**A (n=1359)**	**B (n=109)**	**C (n=277)**	**D (n=1491)**	**E (n=340)**	
**Residence**	**x**^**2**^=40.227*					
Urban	29.2	2.7	7.7	46.5	13.8	702
Rural	40.2	3.1	7.8	40.5	8.4	2874
**Region**	x^2^=221.406*					
Punjab	35.3	1.8	5.9	48.7	8.3	2024
Sindh	49.6	2.6	5.6	32.8	9.4	774
KP	31.2	6.6	13.7	35.5	13.1	564
Baluchistan	39.7	7.5	17.3	23.8	11.7	214
**Economic Status**	x^2^=308.721*					
Poor	49.1	3.1	7.3	33.9	6.6	2020
Middle	31.7	2.3	8.4	46.9	10.7	738
Rich	15.9	3.6	8.3	56.6	15.6	808
**Birth Order**	x^2^=53.158*					
1–2	30.9	3.9	8.0	47.4	9.8	1033
3–4	36.0	3.2	7.5	42.9	10.4	1023
5 and Above	44.2	2.4	7.7	36.9	8.7	1520
**Total (%)**	**38.0**	**3.1**	**7.8**	**41.7**	**9.5**	**100.0**

(A) Cost too much; (B) Facility too far; (C) Not customary; (D) Not necessary; (E) Facility closed * 5% level of significance.

Similarly, 44% of Women having higher birth order (5+) reported that they prefer home deliveries due to high costs at health facilities. Interestingly, however, only 2% women reported that health facility is too far from their residence and transport is not available to go there. Socio-cultural factors remain least reported reason in determining the choice of place of delivery across the categories but the number is a little higher for Khyber Pukhtunkhwa and Baluchistan where 13.7% and 17.3% women deliver at home respectively being it customary.

### Bivariate analysis

A significant association is observed between all major socio-economic and demographic characteristics of mother and choice of place of delivery. Results are suggestive that, in Pakistan, 63%total births are delivered at home. It is evident from Table 3 that, about 72% (p_≤_0.000) women from rural area delivered their babies at home rather than health facilities. Nearly 82% poor (p_≤_0.000), 70% (p_≤_0.000) women belonging to higher age group (35–49), and almost 81% women residing in Baluchistan are found delivering babies at home. Regarding socio-economic perspective of the respondent, 75% women with no formal education are found having deliveries at home instead of health facility. We also found that81% (p_≤_0.000) women working in agriculture sector delivered babies at home. Nearly 75% (p_≤_0.000) of women who have 5 and more children and almost 77% (p_≤_0.000) those who do not discuss pregnancy related issues with their husbands have deliveries at home rather than availing the health facility.

**Table 3:** Percent Distribution of women who are having deliveries at home by their background characteristics

**Characteristics**	**Place of delivery %**		**P-Value**	**N (5677)**
	**Health Facility**	**Home**		
**Residence**				
Urban	59.1	40.9	0.000	1715
Rural	27.5	72.5		3962
**Province**			0.000	
Punjab	36.4	63.6		3182
Sindh	44.9	55.1		1405
KP	31.8	68.2		827
Baluchistan	18.6	81.4		264
**Economic status**			0.000	
Poor	18.2	81.8		2483
Middle	32.8	67.2		1099
Rich	61.5	38.5		2095
**Education level of woman**			0.000	
No education	24.6	75.4		3668
Primary to middle	47.7	52.3		1207
Above middle	77.4	22.6		801
**Education level of husband**			0.000	
No education	22.7	77.3		2007
Primary to middle	34.8	65.2		1692
Above middle	53.6	46.4		1959
**Woman occupation**			0.000	
Not working	40.5	59.5		4026
Agriculture	18.5	81.5		728
Sales	38.4	61.6		734
Others	28.0	72.0		189
**Age**			0.000	
15-24	38.8	61.2		1334
25-34	39.5	60.5		2952
35-49	30.1	69.9		1390
**Birth order**			0.000	
Less than 3	48.3	51.7		2000
3 – 4	38.0	62.0		1648
5 and above	25.1	74.9		2029
**Discussed with husband about place of delivery**			0.000	
Not discussed	22.5	77.5		3148
Discussed	55.1	44.9		2529
**Total (%)**	**37.0**	**63.0**		

### Multivariate

Model 01 in Table 4 reports the results for the women empowerment as predictor of choice for place of delivery. Education of mother is found to be significant predicator of choice of place of delivery and mothers having education primary to middle level are less likely to deliver at home than those having no education, (p_≤_0.000; OR 0.521; 95%; CI 0.449-0.604) while mothers with education level above middle are nearly 0.2 times less likely to deliver at home (p_≤_0.000; OR 0.183; 95%; CI 0.150-0.225). These results are in concurrence with Wagle et al. [5], Bolam et al. [6]. Compared to women having illiterate husbands, women having educated husbands are 0.60 times less likely to deliver babies at home (OR 0.602; 95%; p_≤_0.0005; CI 0.512-0.708). Probability of delivering baby at home decreases as husbands’ education improves to middle level (p_≤_0.05; OR 0.786; 95%; CI 0.671-0.921). These results are in concurrence with Thaddeus and Maine [4], Shah et al. [7] and Tabatabaie et al. [8].

**Table 4:** Odds Ratio of women, five years preceding the survey, having deliveries at home instead of any heath facility

**Indicators**	**Model-01**	**Model-02**	**Model-03**
**Education level of woman** (No education) ^(R)^			
Primary to middle	0.521**	0.547**	0.683**
	(0.449, 0.604)	(0.470, 0.636)	(0.580, 0.804)
Above middle	0.183**	0.208**	0.334**
	(0.150, 0.225)	(0.170, 0.225)	(0.267, 0.419)
**Education level of husband** (No education) ^(R)^			
Primary to middle	0.786*	0.814*	0.911
	(0.671, 0.921)	(0.694, 0.955)	(0.771, 1.077)
Above middle	0.602**	0.618**	0.757*
	(0.512, 0.708)	(0.525, 0.728)	(0.636, 0.901)
**Woman occupation** (Not working) ^(R)^			
Agriculture	1.556**	1.491**	1.078
	(1.260, 1.922)	(1.205, 1.844)	(0.858, 1.353)
Sales	1.124	1.096	1.037
	(0.936, 1.349)	(0.912, 1.317)	(0.857, 1.255)
Others	1.236	1.185	1.251
	(0.862, 1.772)	(0.824, 1.705)	(0.853, 1.833)
**Discussed with husband about place of delivery** (Not Discussed) ^(R)^			
Discussed	0.342**	0.343**	0.397**
	(0.303, 0.387)	(0.303, 0.388)	(0.348, 0.451)
**Age** (15–24) ^(R)^			
25-34	_	0.833*	0.826*
		(0.705, 0.985)	(0.695, 0.983)
35-49	_	0.655*	0.638**
		(0.525, 0.818)	(0.508, 0.803)
**Birth order** (Less than 3) ^(R)^			
3 – 4	_	1.614**	1.755**
		(1.372, 1.900)	(1.483, 2.077)
5 and above	_	2.204**	2.383*
		(1.830, 2.655)	(1.966, 2.890)
**Province** (Punjab) ^(R)^			
Sindh	_	_	0.534**
			(0.456, 0.626)
KP	_	_	0.781*
			(0.645, 0.947)
Baluchistan	_	_	1.1620*
			(1.131, 2.318)
**Residence** (Urban) ^(R)^			
Rural	_	_	1.501**
			(1.284, 1.755)
**Economic status** (Rich)^(R)^			
Poor	_	_	2.634**
			(2.181, 3.182)
Middle	_	_	1.573**
			(1.314, 1.883)
Constant	5.180	4.026	1.692
Hosmer-Lemeshow test (Significance)	0.108	0.101	0.257
**Total (Weighted)**	**5659**	**5658**	**5658**

*5% level of Significance; **1% level of Significance; ( ) confidence interval at 95%.

Mothers working in agriculture sectors are 1.5 times more likely to deliver at home rather than health facilities (p_≤_0.000; OR 1.556; 95%; CI 1.260-1.922) as compared to women not working. Women empowerment, captured through women discussing the pregnancy related issues with their husband ,has a strong impact on their choice of place of delivery and women discussing with their husbands are 0.34 times less likely to deliver baby at home (p≤0.000; OR 0.342; 95%; CI 0.303-0.387) as compared to their counter parts having no freedom to discuss with husband. Furthermore the fact that mothers who do not discuss place of delivery with their husbands are more likely to deliver babies at home (Lambrechts et al. [9], Campbell and Graham [10], Kerber et al. [11]).

In second specification (model 02, Table 4) control variable including current age of mothers and birth order are added to model 01. Both the variables significantly affect the choice of place for delivering babies. Notably young age mothers (15 to 24) are more likely to take home births as compared to older mothers (Susan Mayor [12]). The birth order of children matters significantly and fifth and later born babies are 2.20 times more likely to be delivered at home instead of health facilities (p≤0.000; OR 2.204; 95%; CI 1.830-2.655) and babies born 3rd to 4th are1.61 time less likely as compared to those having birth order 5 and above (p≤0.000; OR 1.614; 95%; CI 1.372-1.900).

Model-03 concludes the estimations wherein we include some background characteristics indicators such as region, place of residence and wealth status of women. Regional background of mothers enters as significant predicator. Education of mother, despite all controls, remains significant and mothers having education primary to middle level are less likely to deliver at home than those having no education, (p_≤_0.000; OR 0.683; 95%; CI 0.580-0.804). Mothers with education level above middle are nearly 0.3 times less likely to deliver at home as compared to illiterate mothers (p_≤_0.000; OR 0.334; 95%; CI 0.267-0.419). Women having educated husbands are 0.91 times less likely to opt for homebirths as compared to those having illiterate husbands (p_≤_0.000; OR 0.757; 95%; CI 0.636-0.901).

The odds ratio for working status of women registered a decline when controlled for socio-economic variables and mothers working in agriculture sector are slightly more likely to deliver at home rather than health facilities as compared to non-working women (p_≤_0.000; OR 1.078; 95%; CI 0.858-1.353). Women who discuss the place of delivery with their husbands are 0.39 times less likely to deliver at home rather than health facilities as compared to those mother who do not discuss (p_≤_0.000; OR 0.397; 95%; CI 0.348-0.451). Mothers of age 25–34 and 35–49 years are 0.82 times (p_≤_0.05; OR 0.826; 95%; CI 0.695-0.983) and 0.65 times (p_≤_0.000; OR 0.638; 95%; CI 0.508-0.803) less likely to deliver their babies at home as compared to youth age group (15–24) respectively. Fifth and later born babies are 2.38 times (p_≤_0.05; OR 2.383; 95%; CI 1.966-2.890) more likely to be delivered at home as compared to babies with order less than three.

A significant variation is registered across the provinces and mothers belonging to Baluchistan province are 1.16 times (p_≤_0.05; OR 1.1620; 95%; CI 1.131-2.318), more likely to deliver their babies at home as compared to mothers residing in Punjab while mothers belonging to Sindh (p_≤_0.000; OR 0.534; 95%; CI 0.456-0.626 and KP (p_≤_0.05; OR 0.781; 95%; CI 0.645-0.947) are also less likely to deliver the babies at home as compared to Punjab. A plausible explanation for outcome for Baluchistan can be traced into insufficient resources regarding health services and strong cultural barriers. Economic status of mothers enters as significant predictor of the choice of delivery place and it observed that mothers belonging to rich class are less likely to deliver babies at home as compared to poor. Poor mothers are 2.63 times (p_≤Z_0.000; OR 2.634; 95%; CI 2.181-3.182) more likely to deliver babies at home rather than health facility and similar results are reported by Bustreo et al. [13].

## Conclusion

The relatively higher cost of health is a bottleneck in the way of delivering babies at health centers. It is evident from the findings that the majority of the mothers are compelled to opt for home confinement because of their inability to bear high costs attached with hospital/health facility confinement due to poor economic status. Parental education appears to be a significant predictor and increased education levels of parents’ decreases the likelihood of home births. Further, data have shown negative impact of women empowerment on home births supporting the findings of Hussain et al. [14]. One of the interesting findings is that women who discuss with their husbands regarding place of delivery delivered babies at health facility rather than home. As expected, women from rural areas are more likely to have home birth as compared to urban mothers. Education of parents, birth order of children and wealth status of mothers are found to be most important factors influencing the choice of place of delivery. Youth age groups of mothers are vulnerable to delivering babies at home. Poverty driven working, especially in agricultural sector, increases the probability of babies being delivered at home. Mothers belonging to Baluchistan are found having deliveries at home because of strong cultural norms.

The study recommends that education of mothers and husbands should be improved so that practices and attitude regarding reproductive rights and safe delivery care is also improved. Cost effective health facilities must be introduced. Mothers and child health care centers should be increased so that people may have an access to health care services and health facility in Pakistan. Finally, advocacy and awareness campaigns must also be run to raise awareness about benefits of delivering babies at health facilities in terms of maternal and child health.

## Abbreviations

PDHS: Pakistan Demographic and Health Survey; DHS: Demographic and Health Survey; NIPS: National Institute of Population Studies; MCH: Mother and Child Health; UNFPA: United Nations Population Fund; WHO: World Health Organization; PBS: Pakistan Bureau of Statistics; EmONC: Emergency Obstetric and Neonatal Care; KP: Khyber Pukhtunkhwa; OR: Odds Ratio; CI: Confidence Interval.

## Competing interests

The authors declared that they have no conflict of interests.

## Authors’ contributions

Study concept and design: SAJ, MDA, AS; Literature Review: MDA, AS, MIH; Data Analysis: WI, SAJ: Interpretation of data: SAJ, WI, AH; Drafting of the manuscript: SAJ, MDA, WI, AH, FS: Critical revision of the manuscript: SAJ, AH, MIH, FS; Study supervision: SAJ. All authors read and approved the final manuscript.

## Pre-publication history

The pre-publication history for this paper can be accessed here:

http://www.biomedcentral.com/1471-2393/13/137/prepub
